# Implementing a case management intervention for frequent users of the emergency department (I-CaM): an effectiveness-implementation hybrid trial study protocol

**DOI:** 10.1186/s12913-018-3852-9

**Published:** 2019-01-11

**Authors:** Véronique S. Grazioli, Joanna C. Moullin, Miriam Kasztura, Marina Canepa-Allen, Olivier Hugli, Judy Griffin, Francis Vu, Catherine Hudon, Yves Jackson, Hans Wolff, Bernard Burnand, Jean-Bernard Daeppen, Patrick Bodenmann

**Affiliations:** 1 Department of Ambulatory Care and Community Medicine, Vulnerable Populations Center, Lausanne University Hospital, Rue du Bugnon 44, 1011 Lausanne, Switzerland; 2 Faculty of Health Sciences, Curtin University, Perth, Australia; 3 Emergency Department, Lausanne University Hospital, Lausanne, Switzerland; 4 Department of Medicine, Well Cornell College Of Medicine, New York, USA; 5 Department of Family and Emergency Medicine, University of Sherbrooke, Sherbrooke, Canada; 6 Division of primary care medicine, Geneva University Hospitals, Geneva, Switzerland; 7 Division of Prison Health, Geneva University Hospitals and Faculty of Medicine, Geneva, Switzerland; 8 Institute of Social and Preventive Medicine, Lausanne University Hospital, Lausanne, Switzerland; 9 Alcohol Treatment Centre, Lausanne University Hospital, Lausanne, Switzerland

**Keywords:** Frequent users of the emergency department, Vulnerability, Case management, Implementation science

## Abstract

**Background:**

ED overcrowding represents a significant public health problem in developed countries. Frequent users of the emergency departments (FUEDs; reporting 5 or more ED visits in the past year) are often affected by medical, psychological, social, and substance use problems and account for a disproportionately high number of ED visits. Past research indicates that case management (CM) interventions are a promising way to reduce ED overcrowding and improve FUEDs’ quality of life. There is, however, very limited knowledge about how to disseminate and implement this intervention on a large scale to diverse clinical settings, including community hospitals and non-academic centers. This paper describes the protocol of a research project aiming to implement a CM intervention tailored to FUEDs in the public hospitals with ED in the French-speaking region of Switzerland and evaluate both the implementation process and effectiveness of the CM intervention.

**Methods:**

This research project uses a hybrid study design assessing both implementation and clinical outcomes. The implementation part of the study uses mixed methods a) to describe quantitatively and qualitatively factors that influence the implementation process, and b) to examine implementation effectiveness. The clinical part of the study uses a within-subject design (pre-post intervention) to evaluate participants’ trajectories on clinical variables (e.g., quality of life, ED use) after receiving the CM intervention. We designed the study based on two implementation science frameworks. The Generic Implementation Framework guided the overall research protocol design, whereas the RE-AIM (reach, efficacy, adoption, implementation and maintenance) framework guided the implementation and effectiveness evaluations.

**Discussion:**

This research project will contribute to implementation science by providing key insights into the processes of implementing CM into broader practice. This research project is also likely to have both clinical and public health implications.

**Trial registration:**

NCT03641274, Registered 20 August 2018.

## Background

ED overcrowding is a common public-health concern in developed countries impacting both patients and health system outcomes [1]. For instance, there are over 1.4 million annual ED visits in Switzerland (8.5 million inhabitants) with 84% of EDs reporting overcrowding [2]. Frequent users of the emergency departments (i.e., FUEDs; reporting 5 or more ED visits in the past year) [3] are in need of targeted attention from clinicians, public health advisors and researchers. FUEDs disproportionately access ED services, and contribute to ED overcrowding (i.e., FUEDs account for 3 to 8% of all patients and 12 to 28% of all ED visits) [4–6]. Driving this high use of health care services is the fact that FUEDs are often affected by multiple chronic medical diseases (e.g., heart disease, cancer) [7–9], as well as psychiatric, substance use and social problems (e.g., unemployment or social isolation) [4, 10–12]. In response, important research efforts have been dedicated to identify, develop and test interventions tailored to this population, such as case management (CM) intervention. Past research has found CM intervention as a promising way to reduce ED overcrowding and improve FUEDs’ quality of life [3, 12–16]. However, there is limited knowledge about how to disseminate and implement such an intervention on a large scale to diverse clinical settings, including community hospitals and non-academic centers. Implementation science provides a framework for integrating evidence-based interventions, such as the CM intervention, in real-world settings while evaluating their feasibility, acceptability and effectiveness over the implementation process. In this article, we describe the protocol of a study funded by the Swiss National Science Foundation (FNS 407440_167341) that aims to implement a CM intervention for FUEDs in several hospitals in the French-speaking region of Switzerland.

### The evidence-based practice being implemented

In line with FUEDs’ specific needs, the CM intervention aims at redirecting and reorienting FUEDs to a range of services within the hospital and community-based settings to improve the quality of care for these patients and reduce the ED overcrowding that negatively impacts all ED patients [12]. The CM intervention targets several mechanisms through which the CM intervention is expected to decrease FUEDs ED visits and related costs and to increase their quality of life. In addition to cares coordination, these mechanisms include health care empowerment (i.e., being engaged, informed, collaborative and committed to one’s health care) [17], perceived self-efficacy (i.e., the belief in one’s ability to perform a task) [18], and health literacy (i.e., skills to meet the complex demands related to health) [19].

Building on previous findings related to CM effectiveness [12], our research team developed and tested a CM intervention tailored to FUEDs in Lausanne University Hospital [3, 20]. Based on FUEDs needs and characteristics [21, 22] this intervention targets several specific components. First, the case managers provide counseling and education on health care utilization, substance abuse and the social determinants of health related to FUEDs, using skills such as motivational interviewing and cross-cultural competences [23, 24]. Additionally, the case managers enable access to concrete services, such as social services (i.e., providing support on income assistance, stable housing, improved health insurance coverage), and refer FUEDs to psychiatric, substance abuse treatment, and/or medical services (e.g., GP or medical specialist) on a case-by-case basis. Another key element of the CM intervention is to connect all health care or social service providers within the hospital and in the community, promoting continuity of care and improving the FUEDs’ ability to navigate the complex health care system. Along with this key dimension of the CM intervention, case managers send on a case-by-case basis letters summing up their intervention to FUEDs’ general practitioners or other involved health-care providers.

### Case management intervention effectiveness

Previous research among FUEDs has established CM intervention effectiveness in improving housing and environmental quality of life and reducing homelessness [12, 13]. There is however, mixed evidence of CM intervention effectiveness in reducing ED use. On one hand, systematic reviews conducted in 2011 [12] and 2016 [14] on interventions including CM, care navigation, patient education and disease specific management programs, found that CM intervention only consistently reduced frequent ED use. On the other hand, results yielded in the few randomized clinical trials conducted to date are mixed [3, 15, 25, 26]. For instance, a recent randomized clinical trial conducted by our research team tested CM intervention efficacy [3]. FUEDs were randomized to the CM intervention or control groups. After 12 months, participants in the intervention group made 2.71 (+/− 0.23) ED visits versus 3.35 (+/− 0.32) visits in the control group (ratio 0.81, 95% CI 0.63–1.02), corresponding to a 19% absolute reduction in ED use. Of important note however, findings indicated that CM intervention effect on the number of ED visits was not significant. Therefore, although most findings support CM intervention effectiveness in reducing ED use and improving FUED’s quality of life, research further evaluating these questions is needed. Accordingly, we selected a hybrid design for this current study, examining both implementation and clinical outcomes [27].

### The guiding implementation science framework

Numerous conceptual frameworks exist to guide the design of research aiming to implement evidence-based practices in real-world settings. In this study, we used the Generic Implementation Framework [28] to guide the overall research protocol design and the RE-AIM (reach, efficacy, adoption, implementation and maintenance) [29] framework to guide the implementation outcomes evaluation.

#### The generic implementation framework (GIF)

The GIF was developed based on a systematic review of the literature on conceptual implementation frameworks and aimed to provide guidance on the basic components required in implementation [28]. According to the GIF, implementation is a non-linear, iterative process, but may be divided into several stages (i.e., development, exploration, preparation, operation and sustainability). At each stage, specific factors strategies and evaluations influence the implementation process. Specific factors (i.e., influencing factors) include barriers and facilitators influencing the implementation process, such as health-care providers’ attitudes toward the intervention being implemented or organizational readiness to change [30]. Strategies refer to implementation interventions (e.g., clinical staff training) to assist the implementation process, whereas evaluations occur to assess the implementation process and outcomes along with the evaluation of the evidence-based intervention (i.e., the CM intervention) outcomes. Influencing factors, strategies and evaluations depend on the evidence-based intervention being implemented and the context in which it is being implemented. They also vary across settings and throughout stages of the implementation process. Finally, according to the GIF, influencing factors exist at multiple levels (e.g., individual or organizational). Therefore, strategies and evaluations should target numerous levels. We chose the GIF to develop the current implementation protocol to ensure covering the core implementation concepts. Each core component of the GIF was tailored to the current research project, in particular to the CM intervention being implemented and to the settings in which it is being implemented.

#### The RE-AIM framework

In addition to the GIF, we used the RE-AIM framework to guide the implementation and effectiveness evaluations [29]. According to the RE-AIM framework, implementation effectiveness depends on five factors: reach, effectiveness, adoption, implementation and maintenance. Reach refers to the participation rate and representativeness of the population targeted by the intervention (i.e., number of patients receiving the intervention divided by the total number of targeted patients), whereas effectiveness refers to the impact of the intervention (at the patient-level). Adoption relates to the proportion of targeted settings adopting the intervention being implemented. Implementation refers to the fidelity with which the intervention is being delivered in the real-world setting (as compared with the evidence-based intervention as it was originally designed). Finally, maintenance refers to the extent to which an evidence-based practice is integrated and sustained in real-world settings over time. Accordingly, the proposed evaluation embedded in the current study protocol targets these five factors.

### Aims of the study

To the best of the authors’ knowledge, no previous study has focused on the implementation of a CM intervention for FUEDs in Switzerland. French-speaking Switzerland includes 7 cantons and covers 23% of the extension of the country (with 2 million persons in 2015). ED overuse is of practical concern to public health advisors and clinicians. Research focusing on both implementation and clinical outcomes may provide key insights into the mechanisms and processes for disseminating and implementing a CM intervention for FUEDs into broader practice in the real-world settings. This study aims at 1) implementing a CM intervention for FUEDs in public hospitals with EDs in the French-speaking region of Switzerland, 2) studying the process of implementation of the intervention and, 3) studying CM intervention’s effectiveness among FUEDs. Regarding the intervention effectiveness assessment, we expect that FUEDs will evince improvements in clinical outcomes (e.g., higher scores in quality of life) and decreases in ED visits after receiving the CM intervention.

## Methods and design

### Design

This research project features a Type 2-hybrid study design focusing on both implementation and clinical outcomes [27]. The implementation part of the study uses mixed methods a) to describe both qualitatively and quantitatively factors that may influence implementation process and b) to assess implementation outcomes. The clinical part of the study uses a within-subject design (pre-post intervention) to evaluate participants’ trajectories after receiving the CM intervention.

### Setting

All community and academic public hospitals with an adult ED opened 24 h/ 7 days per week throughout the French-speaking region of Switzerland interested in implementing the CM intervention are eligible. There are no exclusion criteria.

### Participants

Patients visiting the ED implementing the CM intervention are eligible if they are ≥18 years, able to communicate in a language that is spoken by the local team or a professional interpreter and have ≥5 ED visits in the past 12 months recorded. Exclusion criteria include presenting less than 2 vulnerability dimensions (i.e., social, somatic, mental, risk behaviors) besides frequent ED use, being unable to give informed consent, planning to stay in Switzerland less than 18 months, not expected to survive at least 18 months, awaiting for incarceration or being currently incarcerated, having a family member already enrolled in the study.

Given that this research study aims to disseminate and implement a CM intervention, we are neither aware of the number of hospitals that will eventually participate, nor of the number of participants who will be enrolled in each hospital.

### The CM intervention

The CM intervention, as developed by our research team, typically includes the following steps. First, health-care providers and/or administrative staff detect FUEDs in the ED through an electronic alert in patients’ files. They then notify the case manager that a FUED is in the ED. Next, if the FUED visiting the ED agrees to do so, the case manager makes a first evaluation of the FUED’s situation in the ED, aiming to identify his main problems, the involved health-care network and the priorities that need to be addressed. After this first contact and if the FUED agrees ambulatory care, the case manager writes an intervention report and sets an appointment with the patient. Notably, case managers send on a case-by-case basis letters summing up their intervention to FUEDs’ general practitioner or other involved physicians. Ambulatory care consists of setting a number of appointments with the case manager on a case-by-case basis (in the ED, in the community or health-care network, or at the patient’s home depending on situation and needs). Concretely, the case manager, together with the patient, identifies objectives based on the patient’s specific needs, which will define the total number of appointments needed. Ambulatory care aims at reaching these objectives through appointments with the involved health-care network and referral to specialized services fitting the patient’s needs. Staff involved in this step include the case manager, specialized workers from the health-care network, and sometimes, the patient’s relatives. Whenever possible, the patient is asked to be active in the decisions taken to address his social and medical needs (patient empowerment). The intervention concludes when the patient is integrated into a coordinated and functional health-care network.

### Study procedure, implementation strategies and ethics

The whole project should be completed in 3.5 years over five phases (see Fig. 1). We present below the study procedure over the five phases. Table 1 presents a summary of the implementation strategies by phase. We will use hereafter the term I-CaM research team to refer to staff working on the research project in the home institution, including 2 research assistants (bachelor-level students in Medicine or Social Sciences), a research nurse (nurse, MPH), a scientific collaborator (Ph.D), and the research project leader (Associate Prof, MD, MSc).

**Table 1:** Description of the Implementation Program Components by Phase

Implementation program components	Description	Implementation phase	ED staff involvement
Needs and interest assessment	I-CaM team sends a survey to all eligible hospitals to assess health-providers needs and interests regarding the CM intervention.	Exploration	10 min
Hospital orientation	I-CaM team meets with key ED staff to provide more information about the CM intervention and the study procedures.	Exploration	2 h
Workshop	Interested key ED staff participate in a one-day workshop aiming to provide basic training on the CM intervention and assist in their decision of whether to adopt the CM intervention	Exploration	1 day
CM toolkit	The CM toolkit includes a detailed description of the CM intervention components, a validated framework of vulnerability among FUEDs to use as a screening tool and a booklet with practical information to use as models for the implementing sites.	Preparation, operation and sustainability	Continued use
Team member selection support	Provides a description of key team member characteristics to support team member selection at implementing sites	Preparation	Continued use
Site visits & training	I-CaM team meets with key staff in implementing sites to provide support to establish available resources on-site, to tailor the CM intervention to local realities and to provide training and coaching regarding the CM intervention to the full CM team	Preparation	2 visits of 2 h
Coaching, technical assistance & feedback	The I-CaM coach (I-CaM research nurse) conducts site visits every three months with as needed support to provide tailored assistance and feedback to each site	Operation	Between 4 and 8 h depending on sites’ needs
Coaching	The I-CaM coach provides support and technical assistance upon request from implementing sites	Sustainability	Depending on sites’ needs

#### The development and exploration phases

The I-CaM research team will develop a CM Toolkit, the team member selection-support materials and the informational announcement to be disseminated to eligible hospitals. The I-CaM research team will also develop and send by email a survey aiming to gauge interest and needs regarding the CM intervention to all eligible hospitals (to head physicians of EDs). The survey will include around 20 closed and opened-ended items addressing awareness, perceptions and knowledge of the FUED problematic as well as specific needs and interest regarding the CM. For instance participants will be asked to indicate how often they faced FUEDs over the past 2 years using a Likert scale ranging from 0 = never to 5 = every day, or to describe the main FUED-related difficulties they encounter. Results will be analyzed using both descriptive statistics and qualitative content analysis. Based on the survey results, the I-CaM research team will reach out to the interest hospitals’ key staff to provide more information about the CM intervention and the study procedures. After the meeting, key hospital staff being still interested in the CM intervention will participate in a one-day workshop at the home institution, during which they will receive basic training on the CM intervention, motivational interviewing [24], cross-cultural competencies [23] and on the study procedures, complete a questionnaire and participate in a semi-structured interview. After the workshop, all sites deciding to adopt the CM intervention will be eligible to participate in the study. Finally, hospitals not interested in participating in the study will be proposed participate in a semi-structured interview aiming to evaluate barriers to CM intervention implementation (face-to-face or by phone, depending upon possibilities).

#### The preparation phase

Sites included in the study will prepare for implementation of the CM intervention using the CM toolkit they received at the one-day workshop; with I-CaM research team support, sites will first identify CM intervention team members, including strategic and operational champions and case managers. The champions will a) promote the implementation project within the hospital, b) support its implementation and application and, c) supervise case managers who will be in charge of the CM intervention administration. Next, available resources will be established at each site and data collection systems will be finalized to gather data on implementation and clinical outcomes. The I-CaM research team will also conduct clinical and research trainings (i.e., on the informed consent process and data collection required for the research) to case managers. Finally, at the end of the preparation phase, champions and clinicians involved in the project on-site will complete a questionnaire.

#### The operation phase

The CM intervention will be implemented at all sites included in the study. First, case managers will conduct a screening process to identify eligible participants. At each site, patients fulfilling the inclusion criteria will be contacted by the case managers. The number of participants included will depend upon each site resources. We expect that each site will include at least 2 participants per week on average. The maximum number of inclusions per week per site will be discussed with the research nurse (from I-CaM staff) in charge of the clinical training and supervision in order to avoid inducing work overload. If possible (depending upon resources on each site), eligible patients having been treated in the ED in the 10 days prior the recruitment window will be contacted by phone and proposed to come back to the ED to participate in the study. Remaining FUEDs (i.e., the FUEDs not invited to participate in the study) will receive usual care.

When first meeting the participant, the case managers will present the study and examine the exclusion criterion. If the patient does not have any exclusion criteria and is interested in participating, the case managers will conduct the informed consent process. After providing written consent, participants will receive the CM intervention. After each inclusion on site, the case managers will up-date the I-CaM research team in charge of the clinical assessment (i.e., baseline and follow-up assessments of the health-related variables to describe participants’ trajectories). The I-CaM research team will contact participants and conduct the baseline assessment within 10 days following the inclusion. Participants will then complete follow-up assessments at 3, 6 and 12 months post-baseline. All assessment interviews will be conducted by phone by bachelor-level students (i.e., of medical or social sciences) who will be trained and weekly supervised. For all patients, incentives (i.e., gift cards of 10, 20 and 20 CHF for the 3, 6 and 12-month follow-ups, respectively) and reminders will be used to compensate participation and obtain good retention rates. Other clinical variables (see measures section) will be directly extracted from medical records on-site by the I-CaM research team. Each site implementing the CM intervention will monitor their activities (e.g., tracking the number of eligible patients and the number who received the CM intervention). At the end of the operation phase, the research team will conduct semi–structured interviews with case managers and champions. Both champions and case managers will also complete a questionnaire.

#### The sustainability phase

Following the implementation of the CM intervention, the individual sites will continue the CM programs at their discretion. The research team will monitor the activity at each site and will be available for as-needed support to all sites. Clinical outcomes (i.e., ED use, quality of life etc.) will no more be measured. Finally, at the end of the sustainability phase, case managers and champions will participate in a final semi–structured interview with I-CaM team and complete a last questionnaire.

### Measures

#### Implementation-related measures

Table 2 summarizes the implementation measures by phase. Implementation-related measures will target both the implementation process evaluation (i.e., aiming to describe the process of the implementation and its influencing factors) and the implementation effectiveness evaluation (i.e., the extent to which the implementation was successful) and will include both quantitative and qualitative approaches.

**Table 2:** Summary of the Implementation Measures by Phase

Components	Tool	Participants	Phase
Implementation process evaluation			
Implementation process	Stages of Implementation Completion (SIC)	Champions, case managers, I-CaM team	All
Implementation time-efforts and costs	Stages of Implementation Completion (SIC)	Champions, case managers, I-CaM team	All
Influencing factors			
ED staff awareness and interest in FUED and CM	Online survey	ED staff (public hospitals in the French-speaking Switzerland)	Exploration
	Semi-structured interview	Champions	Exploration
Acceptability, perceived appropriateness and feasibility of the CM	Acceptability Intervention Measure (AIM) and Intervention Appropriateness Measure (IAM) Feasibility of the Intervention measure (FIM)	Champions and case managers	Preparation and operation
	Semi-structured interview	Champions	Exploration
	Semi-structured interview	Champions and case managers	Operation
Intentions to use the CM	Measure of Innovation Specific Implementation Intentions	Champions and case managers	Preparation, operation
Implementation climate	Implementation Climate scale (ICS)s	Champions and case managers	Preparation, operation
	Semi-structured interview	Champions and case managers	Preparation, operation
Readiness for change	Organizational Readiness for Implementation Change (ORIC)	Champions and case managers	Preparation, operation
Implemention effectiveness			
Adoption rate (hospitals included/invited)	Monitoring of the activity on site	Hospitals	Exploration
Reach (patients receiving the CM/eligible)	Monitoring of the activity on site	Hospitals	Operation, sustainability
Implementation effectiveness			
Clinical effectiveness	Clinical outcomes (see Table 3)	Patients receiving the intervention	Operation
Fidelity of the CM	Fidelity checklist	Case managers	Operation and sustainability
Integration of the CM	NoMad survey	Champions and case managers	Operation, sustainability
	Semi-structured interview	Champions and case managers	Operation and sustainability
Normalization of the CM	Measure of Inner Context Sustainment (MICS)	Champions and case managers	Operation and sustainability
	Semi-structured interview	Champions and case managers	Operation and sustainability

##### Quantitative measures assessing implementation process

We will use an adapted Stages of Implementation Completion tool SIC [31] to assess implementation process by tracking the time of achievement of key implementation milestones over the whole implementation process. The SIC is a quasi-quantitative tool that measures progression of implementation activities (e.g., when engagement, provision of services and consultation begin) organized in eight stages, by recording the dates implementation activities were completed. The measure yields three main scores, a) the number of stages completed, b) the time spent in each stage and, c) the proportion of activities completed in each stage. Similarly, we will adapt and use the SIC to evaluate implementation time-efforts and costs by assessing the costs at each stages of the implementation process [32]. Specifically, the SIC will be used to track both hours put forth among staff involved in the implementation project on sites (e.g., case managers) and hours put forth from I-CaM; staff related to the implementation strategies. The measure yields full time equivalent and averaged salary costs scores. The SICs will be used over the whole implementation project.

Influencing factors will be evaluated among champions and case managers throughout the implementation process. As previously mentioned, in the exploration phase we will send an online survey to all ED services in the French-speaking Switzerland to evaluate ED staff’s interest and awareness related the FUED problem and CM intervention as well as their perceptions of needs regarding a CM intervention within their service. Additional influencing factors will be assessed at the end of the exploration, preparation and operation phases with the Acceptability Intervention Measure (AIM), the Intervention Appropriateness Measure (IAM), the Feasibility of the Intervention Measure (FIM) [33] and the Measure of Innovation Specific Implementation Intentions (MISII) scale (see Tables 2 and 3) [34]. Remaining quantitative measures at the preparation and operation phases include the Implementation Climate Scale (ICS) [35] and the Organizational Readiness for Implementation Change (ORIC) [36].

**Table 3:** Summary of the Clinical Measures in the Operation Phase

Components	Tool	Timing
Descriptive variables and covariates		
Demographics	Self-reported questionnaire	Baseline
Vulnerability determinants	Extracted from medical records	Baseline
Clinical outcomes		
ED visits	Extracted from medical records	Baseline, 6, and 12 months
Health-care reorientation	Extracted from medical records	Baseline, 3, 6, and 12 months
Quality of life	WHOQOL-BREF	Baseline, 3, 6, and 12 months
Empowerment	Health Care Empowerment Inventory	Baseline, 3, 6, and 12 months
Self-efficacy	Self-Efficacy Scale	Baseline, 3, 6 and 12 months
Health literacy	European Health Literacy Project Questionnaire	Baseline, 3, 6 and 12 months
Problematic alcohol use	AUDIT-C	Baseline, 3, 6 and 12 months
Precursors of alcohol use	Visual analog rulers assessing importance, intentions, readiness and confidence regarding alcohol use changes	Baseline, 3, 6 and 12 months

##### Quantitative measures assessing the implementation effectiveness

The adoption rate at the conclusion of the exploration phase (i.e., the number of hospitals included in the research project) will be yielded by dividing the number of hospitals included in the research project by the number of hospitals invited to participate. At the operation and sustainability phases, reach will be yielded by dividing the number of patients receiving the CM intervention by the total number of eligible patients. Effectiveness will be examined using clinical outcomes assessed among patients receiving the CM intervention (see measures below). At the operation and sustainability phases, we will also examine the extent to which the intervention is provided as intended (i.e., fidelity to the CM intervention), using a checklist developed by the I-Cam research team of the core components of the CM intervention. This will be self-reported by the case-managers. Finally, we will examine integration and normalization of the CM intervention using the NoMad survey [37] and the measure of inner context sustainment (MICS) (in development and testing).

##### Qualitative measures

Implementation process will be further evaluated qualitatively during semi-structured interviews with champions and case managers examining influencing factors related the outer context (e.g., funding) and the inner context (e.g., organizational readiness to change, receptive context) [38, 39]. Finally, semi-structure interviews will also further examine the CM intervention normalization and integration [40], staff experience with the CM intervention training and coaching, and staff experience regarding the CM intervention implementation and delivery [41].

#### Clinical measures

As described above, the CM intervention aims at reducing ED visits and improving quality-of-life by targeting specific mechanisms, including empowerment, perceived self-efficacy, health literacy and motivation to change. Accordingly, participants will be assessed at baseline and at follow-ups on these clinical variables. Main outcomes will include ED use and quality of life, whereas empowerment, perceived self-efficacy, health literacy and motivation to change will serve as secondary outcomes. Please see Table 3 presenting a summary of clinical measures.

##### Demographic variables

Demographic variables (e.g., biological sex, age) will be assessed at baseline and will serve to describe the sample. They will also serve as covariates in the main analyses.

##### Vulnerability determinants

Vulnerability determinants refer to dimensions that have been found to be positively related to FUEDs [21]. Vulnerability determinants include social factors (e.g., lack of employment, limited social support), somatic factors (e.g., lower perceived health-related quality of life), mental factors (e.g., chronic mental disease), risk behaviors (e.g., alcohol and other drug use) and health-care use (e.g., frequent ED use). Vulnerability determinants will be assessed during the case management; they will therefore be extracted from medical records at baseline and will serve to describe the population and as covariates in the main analyses.

##### ED visits

Number of ED visits over the past year will be extracted from medical records at baseline and at 12-months. Furthermore, ED visits over the past 6 months will be assessed at baseline, 6- and 12 months. ED visits at baseline will serve as covariate in the analyses, whereas the remaining measures will serve as outcome in the mains analyses.

##### Health-care re-orientation

Health-care re-orientation will be extracted from the CM intervention medical records at 12 months. Health-care reorientation at 12 months will be used as outcome.

##### Quality of life

We will use the World Health Organization’s WHOQOL-BREF [42] to evaluate quality of life at baseline, 3-, 6- and 12-month follow-up.

##### Empowerment and self-efficacy

We will evaluate empowerment and self-efficacy at baseline, 3-, 6- and 12 months, with the Health Care Empowerment Informed, Committed, Collaborative and Engaged subscales of the Health Care Empowerment Inventory [17] and Self-Efficacy with the Self-Efficacy Scale [43].

##### Health literacy

Similarly, we will assess health literacy at baseline, 3-, 6- and 12 months using the European Health Literacy Project Questionnaire (HLS-EU-16) [44].

##### Problematic alcohol use and precursors of alcohol use changes

We will measure problematic alcohol use with the AUDIT-C [45]. In addition, we will examine precursors of alcohol use changes (among participants scoring ≥4 for males and ≥ 3 for females at the AUDIT-C) with four single-item visual analog rulers assessing importance, intentions, readiness and confidence regarding alcohol use changes developed by Walton and colleagues [46]. These measures will be assessed at baseline, 3-, 6-, and 12-months.

### Data analysis plan

#### Implementation measures analysis

Descriptive statistics will be used to describe participants’ characteristics and to report implementation outcomes. We will also test implementation measures changes over time. Given that we will have a small sample size regarding implementation outcomes, these analyses will be triangulated with the qualitative data. Specifically, interview contents will be transcribed and explored to identify participants’ recurring codes and categories; we will use conventional content analysis (i.e., a systematic process of coding and classification) [47] using a qualitative software (i.e., Atlas.ti or NVivo) to examine qualitative data.

#### Clinical outcomes

Data will be screened for missing cases, outliers, and normality of distributions using descriptive statistics and plots. We will take appropriate steps to deal with missing data. First, we will conduct analyses to detect missingness patterns and test whether they may be considered “ignorable.” [48] If more than 5% of outcome data are missing, [49] we will divide the sample into 2 groups (i.e., missing, not missing), and use fully observed variables to predict missingness on the affected outcome. If fully observed variables are not significant predictors, missingness may be considered as “observed at random” and fulfilling some criteria for “missing completely at random” (MCAR) assumptions. In that case, we will use multiple imputations procedures for measured outcomes and direct maximum likelihood estimation for structural models. If data missingness is non ignorable (MNAR), we will use pattern-mixture models with multiple imputation to model the missingness mechanism.

Main analyses will comprise multilevel models (i.e., MLM; mixed effects model) [50] utilizing appropriate distributions for the outcome variables (e.g., Poisson, negative binomial, normal). MLM examine the effect of time (after receiving the CM intervention) on clinical outcomes (e.g., quality of life, self-efficacy). MLM is appropriate to handle nonindependence data. Specifically, data will be clustered by participants (i.e., repeated measures) and by hospitals. MLM does not assume independence of observations. Dependence is modeled through random effects (representing different sources of variability in the data). We will include sources of random variability at the group level accounting for between-group differences and another random effects for the individual accounting for within-person differences in the repeated measures. MLM will be adjusted for demographic variables (i.e., age and gender), ED use, at-risk behaviors (e.g., alcohol use disorders) and health status. Descriptive statistics will be conducted on SPSS and MLM on STATA. The significance level will be set at *p* = .05.

## Discussion

This paper describes the protocol of a research project aiming to implement a CM intervention tailored to FUEDs in hospitals in the French-speaking region of Switzerland, using a hybrid design that allows evaluating both implementation and clinical outcomes [27].

### Significance

This project addresses a critical area of ED health services overuse using a practical solution, while taking an innovative research approach that will contribute to the growing literature on this topic. In terms of research feasibility and innovation, implementation science is an emerging field and serves as an ideal theoretical foundation to complement the study of the key clinical outcomes in diverse settings. The hybrid study design, and rooting our project in the established GIF and Re-AIM frameworks, provides a novel and feasible research approach. Findings of the current research project will provide useful information related to the mechanisms and processes for implementing a CM intervention for FUEDs into broader practice in the real-world settings.

This research project is also likely to have social and economic implications. ED overuse and overcrowding is a critical problem, and this project promotes a potential solution. At the Lausanne University Hospital ED, FUEDs accounted for 4.4% of ED patients yet 12.1% of all ED visits (i.e., in 2008–2009; *n* = 5813) [4]. There is a strong link between chronic disease and frequent ED use, driven largely by the medical and social complexity of these patients. Addressing ED overuse through CM redirects FUEDs to more appropriate forms of care, including primary care or substance abuse treatment, while remaining cost-effective. By developing a CM intervention program and studying its implementation at multiple sites throughout the French-speaking region of Switzerland, this project will provide insights and lessons for the broader use of CM interventions for health care services overuse throughout Switzerland.

Finally, the CM intervention targets a defined population— FUEDs —that is frequently highly vulnerable in terms of medical complexity, suffering from multiple chronic medical conditions, mental health and substance abuse treatment needs, as well as modifiable social determinants of health, including homelessness or low household income. Therefore, this project also addresses a medically and socially complex patient population with chronic health care and social service needs, as well as emphasizing improved communication and care coordination within interdisciplinary care teams.

### Limitations

There are several expected limitations in this current research project that were carefully considered while designing the study. Regarding clinical outcomes, the design of this study allows patients’ evolution to be evaluated after receiving the CM intervention (i.e., pre-post intervention; within-subjects design) in the sites implementing the intervention. A well-known limitation related to this design is regression to the mean. In fact, we previously conducted research using gold-standard randomized controlled trial to test the CM intervention efficacy [3] and the proposed within-subjects’ design is consistent with Stages I and II treatment development [51]. That said, interpretation of pre-post intervention changes on health outcomes will be made with caution. Next, self-reported data can be subject to reporting bias [52, 53]. It can however, be reliable when measures are developed for and piloted on the target population, timeframes are manageable, confidentiality is ensured and the target behavior is not stigmatized [54, 55]. The above conditions were carefully considered when designing the study, thereby minimizing the risk of self-report bias. Furthermore, recruitment and retention can be a challenge with the target population (i.e., FUEDs), resulting in missing data. However, previous work with a similar population, has demonstrated the possibility to attain acceptable 1-year retention rates [3]. Procedures for maximizing study retention will therefore be inspired from the latter study. For instance, we will use incentives to help retention over time (gift cards of increased value for each follow-up assessment completed: 10, 20 and 20 CHF for 3, 6 and 12-month follow-up assessment). Additionally, specific steps will be taken to address any bias that may occur because of missing data as described previously. Finally, although not known at this stage, the patient sample size will be larger than the hospital sample size; therefore, the implementation outcomes analyses will not have the same level of analysis and will mostly remain descriptive. To address this limitation, we will use mixed methods (i.e., qualitative and quantitative methods) to assess the implementation process and outcomes.

## Conclusion

FUEDs represent a vulnerable population multiply affected by medical, psychological, social and substance-use problems accounting for a disproportionally high number of ED consultations. CM intervention tailored to this population has been found to be generally effective in reducing ED overcrowding and improving FUEDs’ quality of life [3, 12–15]. There is, however, very limited knowledge about how to translate and implement such an intervention on a large scale to diverse clinical setting, including community hospitals and non-academic centers. In response, this research project aims to implement the CM intervention in several hospitals in French-speaking Switzerland while evaluating both implementation and clinical outcomes, thereby providing key insights into the mechanisms and processes for disseminating and implementing this intervention into broader practice.

## Abbreviations

CM: Case management
ED: Emergency department
FUED: Frequent users of the emergency department
